# Marine Collagen Peptides from the Skin of Nile Tilapia (*Oreochromis niloticus*): Characterization and Wound Healing Evaluation

**DOI:** 10.3390/md15040102

**Published:** 2017-03-30

**Authors:** Zhang Hu, Ping Yang, Chunxia Zhou, Sidong Li, Pengzhi Hong

**Affiliations:** 1 Department of Chemistry, College of Chemistry and Environment, Guangdong Ocean University, Zhanjiang 524088, China; sidongligdou@163.com; 2 College of Food Science and Technology, Guangdong Ocean University, Zhanjiang 524088, China; gdouqyx@126.com (P.Y.); chunxia.zhou@163.com (C.Z.)

**Keywords:** marine collagen peptides, Nile tilapia (*O. niloticus*), characterization, wound healing

## Abstract

Burns can cause tremendous economic problems associated with irreparable harm to patients and their families. To characterize marine collagen peptides (MCPs) from the skin of Nile tilapia (*Oreochromis niloticus*), molecular weight distribution and amino acid composition of MCPs were determined, and Fourier transform infrared spectroscopy (FTIR) was used to analyze the chemical structure. Meanwhile, to evaluate the wound healing activity, in vitro and in vivo experiments were carried out. The results showed that MCPs prepared from the skin of Nile tilapia by composite enzymatic hydrolysis were composed of polypeptides with different molecular weights and the contents of polypeptides with molecular weights of less than 5 kDa accounted for 99.14%. From the amino acid composition, the majority of residues, accounting for over 58% of the total residues in MCPs, were hydrophilic. FTIR indicated that the main molecular conformations inside MCPs were random coil. In vitro scratch assay showed that there were significant effects on the scratch closure by the treatment of MCPs with the concentration of 50.0 μg/mL. In the experiments of deep partial-thickness scald wound in rabbits, MCPs could enhance the process of wound healing. Therefore, MCPs from the skin of Nile tilapia (*O. niloticus*) have promising applications in wound care.

## 1. Introduction

With the quickening pace of life and the change of people’s way of life, the incidence of burns has been increasing today, and burns cause tremendous economic problems associated with irreparable harm to patients and their families [1,2]. In burn care, a variety of drugs, such as silver sulfadiazine and mafenide acetate solution, have been used. However, these drugs have some major disadvantages, such as serious side effects, poor treatment effects for deep burn wounds, clear scar formation and high costs [3,4]. Therefore, it is still essential to develop some novel efficient agents for treatment of burns to meet the urgent demands for clinical application.

Marine collagen has been isolated from many marine sources such as marine fishes [5,6], sponges [7,8,9,10,11] and mollusks [12,13,14,15]. In marine fishes, the fish tissues, including the skin, bone and scale, account for approximately 30% of the processing waste [16]. Marine collagen peptides (MCPs) are derived from marine collagen by chemical and enzymatic hydrolysis [17]. Compared with marine collagen, MCPs have lower molecular weights resulting in easily being absorbed and strong affinities for water [18]. Due to the special marine ecological environment, such as high pressure, low temperature and high salinity, MCPs from marine fishes differ greatly from those from terrestrial livestock in both physicochemical properties and amino acid compositions, and have unique physiological functions including antibacterial [19], antioxidant [16,20], antihypertensive [21,22,23], neuroprotective [24] and anti-skin-aging activities [25]. It was reported that oral administration of marine collagen peptides from Chum Salmon (*Oncorhynchus keta*) skin enhanced cutaneous wound healing and angiogenesis in rats [26]. Most recently, the electrospun tilapia collagen nanofibers which could accelerate skin wound healing rapidly and effectively in the rat model were developed [27]. To our knowledge, however, few reports offered information concerning wound healing activity of tilapia collagen peptides. Earlier, we reported that acid-solubilized collagen was successfully extracted from the skin of Nile tilapia (*O. niloticus*) and the characterization was carried out [28]. Continuing with our efforts in search of functional collagen peptides, herein we describe the preparation and wound-healing evaluation of MCPs from the skin of Nile tilapia (*O. niloticus*). The results will hopefully provide a theoretical basis for the development and clinical application of tilapia MCP products.

## 2. Results and Discussion

### 2.1. Molecular Weight Distribution of MCPs

**Figure 1:**
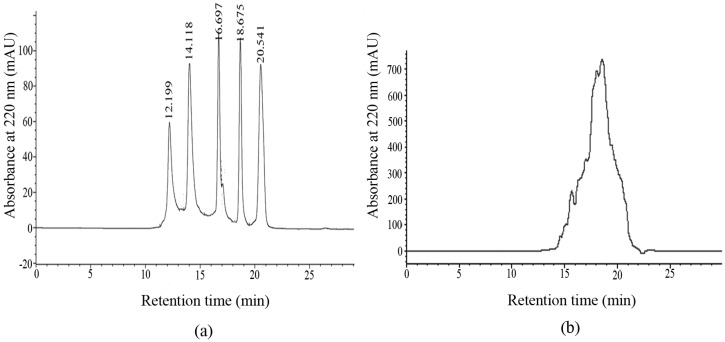
The HPLC chromatograms of (**a**) the standard molecular weight samples and (**b**) marine collagen peptides (MCPs) from the skin of tilapia.

The HPLC chromatogram of the standard molecular weight samples is shown in Figure 1a. With the retention time (*Rt*) as the horizontal axis and lg*Mw* as the vertical axis, the data were fitted into the following regression equation:

lg*Mw* = −0.2263*Rt* + 6.9229 (1)

the value of the determination coefficient (*R*^2^) was 0.9942, which revealed a good linear relationship. The relative molecular weights of the samples could be analyzed based on this linear regression equation. The HPLC chromatogram of MCPs from the skin of tilapia was shown in Figure 1b. The components of less than 1, 3 and 5 kDa accounted for 73.92%, 95.84% and 99.14%, respectively, which showed that MCPs from the skin of tilapia were mainly composed of a number of polypeptides with small molecular weights. Compared with collagen from the skin of tilapia, MCPs possessed better water solubility. This could be explained by the fact that many polar residues in the low-molecular-weight structures of MCPs were exposed to water, resulting in more hydrogen bond formation [29].

### 2.2. Amino Acid Composition of MCPs

MCPs from the skin of tilapia were analyzed in terms of the amino acid composition and the results are shown in Table 1. From Table 1, MCPs from the skin of tilapia contained seven essential amino acids (16.18%) and ten nonessential amino acids (79.56%). Collagen hydrolysates usually contain a high concentration of collagen tripeptides with a Gly-X-Y sequence [30,31]. The contents of glycine, proline and hydroxyproline as the major amino acids in MCPs accounted for 20.92%, 11.32% and 10.28%, respectively. Those were consistent with the gly-pro-hyp sequence. The amino acid contents (proline and hydroxyproline) of MCPs from the skin of tilapia were 200 residues per 1000 total amino acid residues, which were higher than those (between 177 and 184) in acid soluble collagens from the skin and bone of Spanish mackerel (*Scomberomorous niphonius*) [32]. From the amino acid composition, the majority of residues in MCPs were hydrophilic such as glycine, glutamic acid, arginine, aspartic acid, lysine and serine, which accounted for over 58% of the total residues. The hydrophilic property of MCPs could potentially be used to improve histocompatibility.

### 2.3. FTIR Analysis

**Figure 2:**
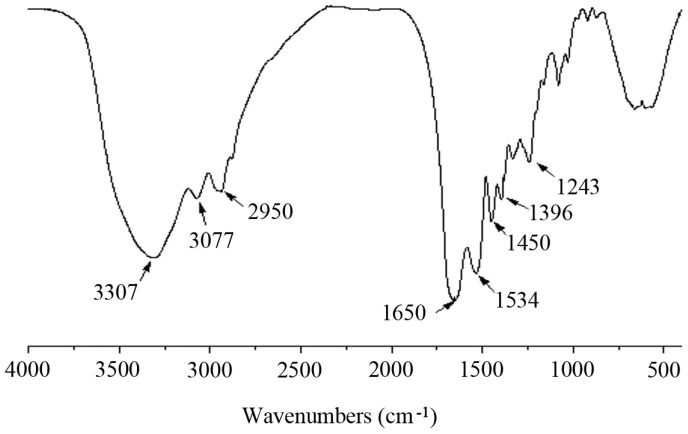
FTIR spectrum of MCPs from the skin of tilapia.

Infrared spectroscopy is sensitive to the chemical structures of molecules and suitable for the determination of proteins and polypeptides under different states, concentrations, and environments and is a useful tool for determining the secondary structure of proteins and polypeptides [33,34]. The infrared spectrum of MCPs from the skin of tilapia was shown in Figure 2. The obvious absorption peaks at 3307 cm^−1^ and 3077 cm^−1^ were typical characteristic amide A and B bands, respectively [34]. The absorption band at 2950 cm^−1^ was attributed to the C-H stretching vibrations. The characteristic absorption peaks of amide I, II and III bands of polypeptides were at 1650, 1534 and 1243 cm^−1^, respectively, which were the characteristic peaks of random coil structure [35]. These results indicated that the main molecular conformations inside MCPs from the skin of tilapia were random coil. The absorption bands at 1450 and 1396 cm^−1^ were ascribed to C-H and O-H bending vibration, respectively.

### 2.4. Effect of MCPs on the Scratch Closure In Vitro

**Figure 3:**
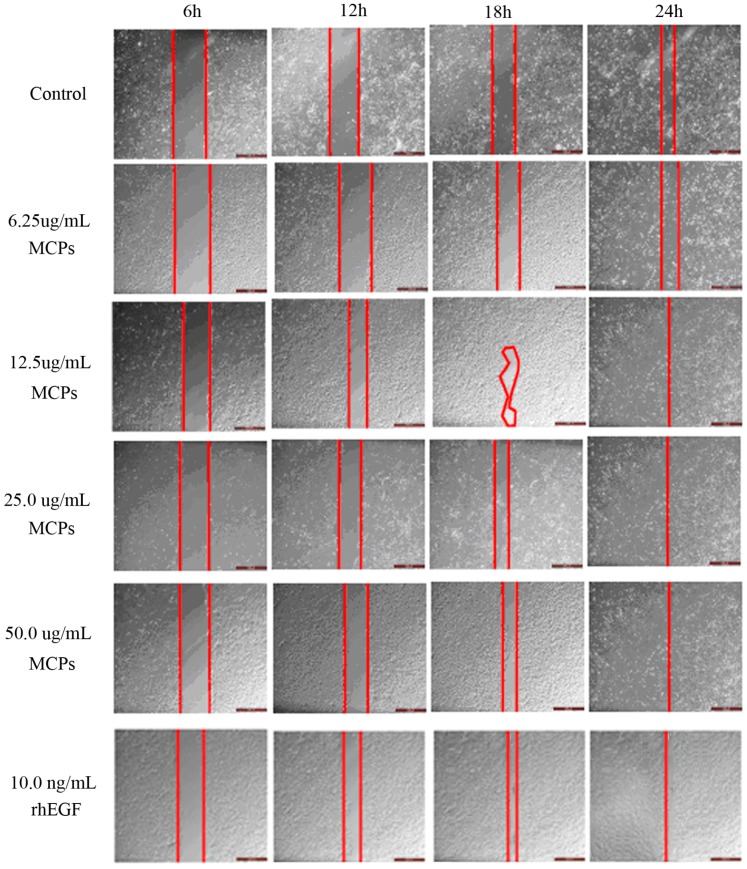
Effect of MCPs from the skin of tilapia on the scratch closure in vitro. Scale bar: 100 μm.

During the process of wound healing, the migration of keratinocyte cells accelerates re-epithelialization process and promotes wound closure [36]. The scratch assay in vitro has usually been used to simulate wound closure [37,38]. Therefore, the effects of MCPs on the healing process were investigated by using in vitro scratch assay with HaCaT cells. The scratch closure rate was calculated at different times and the results were shown in Figure 3. Recombinant human epidermal growth factor (rhEGF, 10.0 ng/mL) potently induced cell migration resulting in wound closure within 24 h. By treatment with MCPs at a low concentration of 6.25 μg/mL for 6, 12, 18 and 24 h, the differences of scratch closure rates were not remarkable compared with the control group (data not shown). At concentrations between 12.5 and 50.0 μg/mL, there were no obvious effects on the scratch closure by treatment with MCPs for 6 h, whereas, significant wound closure effects of MCPs were shown after treatment for 12 h (*p* < 0.05), 18 h (*p* < 0.01) and 24 h (*p* < 0.01) compared with the control group. In particular, the results of 50.0 μg/mL were all highly statistically significant (12 h: 45.52 ± 6.86 vs. 26.38 ± 3.10, *p* < 0.01; 18 h: 70.75 ± 6.86 vs. 49.61 ± 3.56, *p* < 0.01; 24 h: 100.00 ± 0.00 vs. 76.99 ± 3.46, *p* < 0.01). The cell migration induced by 50.0 μg/mL MCPs was almost identical with that by 10.0 ng/mL rhEGF. These results demonstrated that MCPs from the skin of tilapia had outstanding capacity to induce HaCaT cell migration. It is probably the case that the abundant animo acid residues in MCPs provide a suitable environment to induce HaCaT cell migration, although the mechanism of MCPs is not clear.

### 2.5. Wound Healing In Vivo

#### 2.5.1. Scald Model Establishment

**Figure 4:**
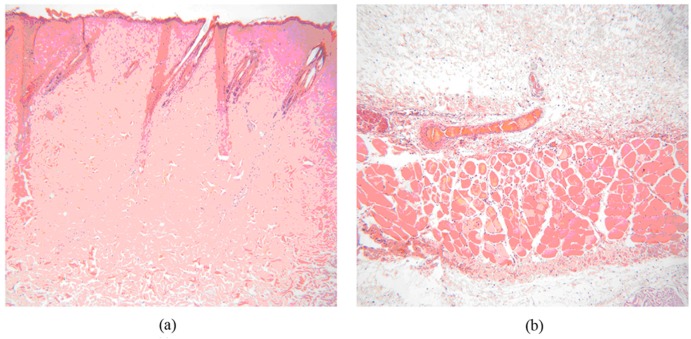
Microscope observation of pathological sections on the post-scald day (H&E, 100×). (**a**) Coagulation necrosis of the epidermis and dermis; (**b**) Impaired skin appendages and focal necrosis associated with inflammatory cell infiltration.

On the post-scald day (PSD), microscopical examination found that coagulation necrosis appeared in the whole epidermis, superficial dermis and parts of deep dermis (Figure 4a). Impaired skin appendages, subcutaneous edema and vascular dilatation were observed in the wounds. Focal necrosis associated with inflammatory cell infiltration of striated muscle cells in the muscular layer had occurred (Figure 4b). In a word, histological features in the wounds meet characters of deep partial-thickness scald, indicating that the deep partial-thickness scald model in New Zealand white rabbits was successfully established.

#### 2.5.2. Effects of MCPs on Scald Wound Healing Rate

The effects of MCPs from the skin of tilapia on wound healing rate in the scalded rabbits were shown in Table 2. In the initial seven days, wound healing rates were negative because of skin edema due to the exudation of tissue fluid after scald, and they increased with no significant differences among the three groups. However, on PSD 11 and 14, there were significant differences between the model control group and MCPs group. Especially on PSD11, wound healing rate of MCPs group (38.8% ± 22.8%) increased more significantly than those of the model control group (8.7% ± 17.2%, *p* < 0.01) and the positive control group (19.5% ± 35.0%, *p* < 0.05). Wound healing rates of MCPs group and the positive control group showed no significant differences on PSD18, 21 and 24, whereas they were significantly higher than those of the model control group (*p* < 0.01). Furthermore, the rabbits in the model control group only achieved 72.1% ± 13.9% wound healing on PSD18, while the rabbits treated with MCPs almost completely healed. These results showed that MCPs from the skin of tilapia had a beneficial effect on wound healing in rabbits.

#### 2.5.3. Histological Evaluation

**Figure 5:**
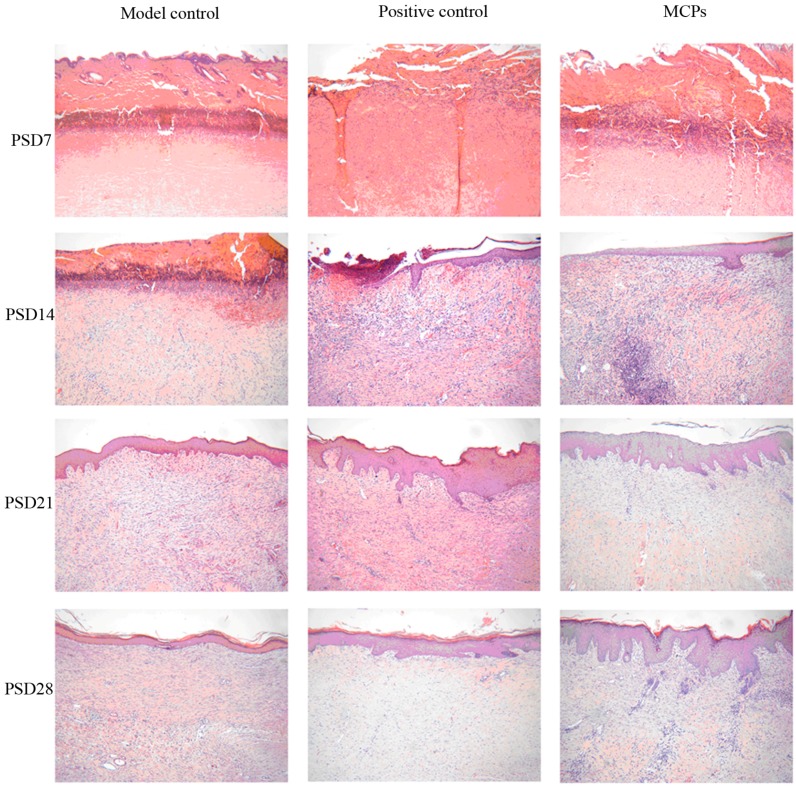
Micrographs of wound tissues in rabbits (H&E, 100×).

The wounds of each group were harvested daily on PSD7, 14, 21 and 28 for histopathological observation (Figure 5). On PSD7, coagulation necrosis of the whole epidermis layer, superficial dermis layer and part of the deep dermis layer as well as significantly impaired skin appendages were observed in the wounds. There were no significant differences among the three groups. On PSD14, few wounds covered by new epidermis and little proliferation of mature granulation tissue were found in the model and positive groups, whereas MCPs group had over half wounds covered by new epidermis and much granulation tissue proliferation in the dermis, indicating that MCPs from the skin of tilapia could facilitate wound healing. On PSD21, compared with the model control group, the positive and MCPs groups showed the appearance of almost wound coverage by new epidermis, active hair follicle proliferation, complete muscular layer structure, fibroblasts and new capillaries. On PSD28, the wounds were completely covered by new epidermis among the three groups. Meanwhile, inflammatory cells disappeared and mature granulation tissue proliferation appeared in dermis layer. However, the formation of scar tissues was seen in the muscle layer between the model control and the positive groups. Overall, histological findings showed that MCPs from the skin of tilapia had beneficial effects on the pathological repair of tissue injury and enhanced wound healing.

Burn wounds are classified as superficial, superficial partial-thickness, deep partial-thickness, full-thickness or subdermal burns by depth. It usually takes three to six weeks or more for the complete healing of deep partial-thickness wound without burn care; moreover, the burns will result in scar formation [4]. Wound healing is one of the most complex biological processes, basically composed of four phases including hemostasis, inflammation, proliferation and remodeling [39,40]. One of the cellular mechanisms is keratinocyte re-epithelialization, which is mainly dependent on keratinocyte proliferation and migration. Cell proliferation can ensure that more cells migrate to the wound and cover it [41,42]. The nuclear factor-κB (NF-κB) is a pivotal mediator in the human immune system and regulates the transcription of a variety of inflammatory mediators. c-Jun NH_2_-terminal kinase (JNK) is predominantly phosphorylated in the cells bordering the wound, indicating that JNK signaling is required for epithelial cells at the wound edge to close the wound [43]. Transforming growth factor-β1 (TGF-β1) is an important factor that plays a key role during wound healing. In every phase of wound healing, TGF-β1 is involved by suppressing inflammatory responses and promoting the formation of granulation tissue [44]. Liu et al. demonstrated that a peptide named AH90 from the frog skin of *Odorrana graham* showed potential wound healing-promoting activity by promoting release of TGF-β1 through activation of NF-κB and JNK mitogen-activated protein kinases signaling pathways [36]. In this study, we found that MCPs from the skin of tilapia could accelerate the healing process and improve the healing effect of skin scald wounds in rabbits, primarily by reducing inflammation, promoting granulation tissue formation, and facilitating rapid proliferation of epithelial cells, endothelial cells and fibroblasts. However, the underlying molecular mechanism remains to be elucidated.

## 3. Materials and Methods

### 3.1. Materials

The skin of tilapia was donated by a local fish processing corporation. The skin was descaled and cut into small pieces and stored at −20 °C for use. Neutral protease (2 × 10^5^ U/g) and papain (6.5 × 10^5^ U/g) were purchased from Pangbo Biological Engineering Co., Ltd. (Nanning, China); Moist scald ointment was purchased from Meibao Pharmaceutical Co., Ltd. (Shantou, China); New Zealand white rabbits were provided by the Guangdong Medical Laboratory Animal Center, Sanshui base (Certificate No. SCXK 20140035, Guangdong, China). The rabbits were individually caged under the conditions of 24 ± 2 °C and 60% ± 10% humidity.

### 3.2. Preparation of MCPs from the Skin of Tilapia

A certain amount of tilapia skin was mixed with water at a solid:liquid ratio of 1:2.5 (*w*/*v*) and then heated. Neutral protease and papain were added when the mixture was heated to 50 °C, and kept for 5 h. Subsequently, the mixture was heated to 100 °C for inactivation, followed by centrifugation. The supernatant was filtered through a 50-nm ceramic membrane. The filtrate was concentrated under reduced pressure and spray-dried into powders.

### 3.3. Determination of Molecular Weight Distribution of MCPs

A high-performance liquid chromatography (Angilent 1200, Palo Alto, CA, USA) was used to analyze the molecular weight distribution of the samples. Acetonitrile/water/trifluoroacetic acid (45:55:0.1) were adopted as the mobile phase, the flow rate was 0.5 mL/min and the UV wavelength was 220 nm. The standard samples consisted of cyyochrome (12,500 Da), aprotinin (6500 Da), bacitacin (1450 Da), ethyl amino acid-ethyl amino acid-tyrosine-arginine (451 Da) and ethyl amino acid-ethyl amino acid-ethyl amino acid (189 Da) were in turn loaded into the column. A standard curve of retention time-absorbance was plotted. The MCPs solution was filtered through a 0.45 μm filter and injected under the same conditions. The molecular weight was calculated according to the retention time using the standard curve equation.

### 3.4. Amino Acid Composition Measurement of MCPs

MCPs from the skin of tilapia were hydrolyzed by dissolving in 6 mol·L^−1^ HCl. The solution was analyzed with an amino acid analyser (S-433D, Sykam, Bremen, Germany).

### 3.5. FTIR Analysis

The Infrared absorption characteristics of MCPs were studied by FTIR spectroscopy (Spectrum 100, PerkinElmer, Waltham, MA, USA). The samples were prepared in potassium bromide disks. The spectra were produced with a wave number range from 4000 to 450 cm^−1^ at a resolution of 4 cm^−1^ over 16 cumulative scans.

### 3.6. In Vitro Scratch Assay

Human immortalized keratinocytes (HaCaT) were cultured in Dulbecco’s Modified Eagle Medium (DMEM, supplemented with 10% fetal bovine serum, 100 U/mL penicillin and 100 μg/mL streptomycin) at 37 °C in an atmosphere with 5% CO_2_. HaCaT cells were split after reaching a confluence of 90%, seeded into 24-well plates at a density of 5 × 10^3^ cells/well and then cultured for 24 h to confluent cell monolayers. A 200-μL pipette tip was used to create a uniform scratch wound on the monolayer of cells. The wounded debris was removed by washing with PBS for twice. The scratch monolayer cells were incubated in serum-free medium containing rhEGF (10.0 ng/mL) as the positive control and MCPs with the varied concentrations from 6.25 to 50.0 μg/mL. The cells without MCPs treatment were used as the blank control. The scratch closure was observed by using a phase-contrast microscope (CKX41-A32PH, Olympus, Tokyo, Japan) and the scratch area was calculated with the Image J software. The scratch closure rate in percentage was obtained by the following formula:

Scratch closure rate (%) = (*A*_0_ − *A_t_*)/*A*_0_ × 100%, (2)

where *A*_0_ is the scratch area at 0 h, and *A_t_* is the scratch area at the designated time.

### 3.7. Effects of MCPs on Skin Scald Wound Healing in Rabbits

#### 3.7.1. Establishment of the Animal Model

Healthy New Zealand white rabbits were anesthetized by intramuscular injection of Sumianxin II (0.5 mL per rabbit) and intravenous injection of sodium pentobarbital (0.6 mL·kg^−1^). After the back hair of rabbits was shaved, one 4-cm^2^ scald wound was produced on both sides of the back using a scalding device (YLS-5Q, Beijing, China). Scalding conditions: scalding head temperature, 100 °C; applied pressure, 1000 g; contact time, 5 s. The deep partial-thickness scald model was established in this way.

#### 3.7.2. Grouping and Treatment

The rabbits were randomly divided into three groups, including model control, positive control and MCPs groups (16 in each group, half male and half female). All the three groups of rabbits were subjected to the deep partial-thickness scald. Moist scald ointment was used as the positive control drug. The model control group was not treated after scalding. The positive control and MCPs groups were treated once daily for 28 days.

#### 3.7.3. Determination of Wound Healing Rate

Wound healing rate was determined with reference to the previously reported method with a few modifications [45]. Briefly, the edges of the wound were drawn on the transparent paper when the wound was covered with a piece of paper, and the shape of the wound was then cut out of the paper and weighed. Wound healing rate was calculated according to the following equation:

Wound healing rate (%) = (*W_i_* − *W_u_*)/*W_i_* × 100%, (3)

where *W_i_* and *W_u_* are the weights of the initial and unhealed wound-shaped paper, respectively, in grams.

#### 3.7.4. Histological Examination

The tissue specimens were harvested after scald treatment for 1, 7, 14, 21 and 28 days, respectively, fixed in 4% formalin, made into paraffin sections, stained with hematoxylin-eosin reagent (HE), and then examined under the microscope for skin structure integrity, the type of cells and granulation tissues.

### 3.8. Statistical Analysis

All the experimental values were expressed as means ± standard deviation (SD). The comparison analysis between the groups was carried out by using the analysis of variance (ANOVA) with the SPSS 21.0, and *p*-values of less than 0.05 were considered to be statistically significant.

## 4. Conclusions

In this study, MCPs prepared from the skin of tilapia by composite enzymatic hydrolysis were composed of polypeptides with molecular weights less than 5 kDa. Infrared spectroscopy showed that the main molecular conformations inside MCPs were random coil. In vitro scratch assay and wound healing experiments of deep partial-thickness scald wound in rabbits indicated that MCPs from the skin of tilapia were an effective and promising agent for burn care. As for the specific molecular mechanisms, we are working for more in depth exploration and the results will be reported in due course.

## Figures and Tables

**Table 1:** Composition and contents of amino acids of MCPs.

Amino Acids	Contains (g/100 g)	Residues Per 1000 Total Amino Acid Residues
Aspartic acid	5.53	48
Threonine *	2.67	25
Serine	3.17	34
Glutamic acid	9.40	81
Glycine	20.92	317
Alanine	9.23	118
Valine *	2.17	22
Methionine *	1.33	10
Isoleucine *	1.33	11
Leucine *	3.18	27
Tyrosine	0.74	6
Phenylalanine *	2.17	15
Histidine	1.01	8
Lysine *	3.33	26
Arginine	7.96	52
Proline	11.32	111
Hydroxy proline	10.28	89
Total	95.74	1000

Note: * essential amino acids.

**Table 2:** The effect of MCPs from the skin of tilapia on wound healing rate (%) in the rabbits ($\bar{x}\pm s$).

Post-Scald Day	Model Control Group	Positive Control Group	MCPs Group
3	−16.4 ± 19.3	−22.7 ± 22.9	−11.8 ± 23.1
7	−7.0 ± 23.1	−1.8 ± 27.5	−3.6 ± 28.6
11	8.7 ± 17.2	19.5 ± 35.0	38.8 ± 22.8 **^,#^
14	56.6 ± 31.1	70.5 ± 23.5	78.6 ± 11.1 *
18	72.1 ± 13.9	95.3 ± 6.4 **	95.9 ± 7.2 **
21	86.2 ± 16.0	98.9 ± 2.0 **	98.0 ± 6.8 **
24	89.8 ± 6.3	100.0 ± 0 **	100.0 ± 0 **
28	100.0 ± 0	100.0 ± 0	100.0 ± 0

Note: * *p* < 0.05 and ** *p* < 0.01 were significantly different compared to the model control group; ^#^ *p* < 0.05 were significantly different compared to the positive control group.
